# NBM-HD-1: A Novel Histone Deacetylase Inhibitor with Anticancer Activity

**DOI:** 10.1155/2012/781417

**Published:** 2011-10-20

**Authors:** Wei-Jan Huang, Yu-Chih Liang, Shuang-En Chuang, Li-Ling Chi, Chi-Yun Lee, Chia-Wei Lin, Ai-Ling Chen, Jing-Shi Huang, Chun-Jung Chiu, Cheng-Feng Lee, Chung-Yang Huang, Chia-Nan Chen

**Affiliations:** ^1^Graduate Institute of Pharmacognosy, College of Medicine, Taipei Medical University, Taipei 110, Taiwan; ^2^School of Medical Laboratory Science and Biotechnology, College of Medicine, Taipei Medical University, Taipei 100, Taiwan; ^3^National Institute of Cancer Research, National Health Research Institutes, Miaoli County, Zhunan 350, Taiwan; ^4^New Drug Research and Development Center, NatureWise Biotech & Medicals Corporation, Nankang, Taipei 115, Taiwan

## Abstract

HDAC inhibitors (HDACis) have been developed as promising anticancer agents in recent years. In this study, we synthesized and characterized a novel HDACi, termed NBM-HD-1. This agent was derived from the semisynthesis of propolin G, isolated from Taiwanese green propolis (TGP), and was shown to be a potent suppressor of tumor cell growth in human breast cancer cells (MCF-7 and MDA-MB-231) and rat glioma cells (C6), with an IC_50_ ranging from 8.5 to 10.3 **μ**M. Western blot demonstrated that levels of p21^(Waf1/Cip1)^, gelsolin, Ac-histone 4, and Ac-tubulin markedly increased after treatment of cancer cells with NBM-HD-1. After NBM-HD-1 treatment for 1–4 h, p-PTEN and p-AKT levels were markedly decreased. Furthermore, we also found the anticancer activities of NBM-HD-1 in regulating cell cycle regulators. Treatment with NBM-HD-1, *p21* ^*(Waf1/Cip1)*^ gene expression had markedly increased while *cyclin B1* and *D1* gene expressions had markedly decreased. On the other hand, we found that NBM-HD-1 increased the expressions of tumor-suppressor gene *p53* in a dose-dependent manner. Finally, we showed that NBM-HD-1 exhibited potent antitumor activity in a xenograft model. In conclusion, this study demonstrated that this compound, NBM-HD-1, is a novel and potent HDACi with anticancer activity *in vitro* and *in vivo*.

## 1. Introduction

Chromatin, a densely packed higher-order complex structure containing DNA and proteins, is present in eukaryotic cells. The functions of chromatin are to package DNA into a small volume to fit in eukaryotic cells and to serve as a mechanism to control gene expression and DNA replication. Major components of chromatin are negatively charged DNA or RNA and other associated proteins especially histones. Histones contain an octamer complex of two copies of H2A, H2B, H3, and H4 proteins, which are very basic because of a richness of the positively charged amino acids such as Arg and Lys [1, 2]. Within nuclei of eukaryotic cells, regulation of gene expression occurs through a dynamic process between two different forms of heterochromatin and euchromatin [3, 4]. In general, heterochromatin is highly compact and shuts down or inactivates gene expression. In contrast, euchromatin is loosely packed and is more accessible to RNA polymerases in order to regulate gene expression. Posttranslational modifications of chromatin, such as acetylation or deacetylation at Lys residues, methylation at Lys or Arg residues, and phosphorylation at Ser resides, are important in gene regulation [5]. All these chemical modifications of amino acids occur on the N-terminal tail of histones [6].

Structural modifications of histones mainly occur via acetylation or deacetylation of the N-terminal tail which affects chromatin remodeling and modulation of gene expression [7, 8]. The states of acetylation and deacetylation of histones are controlled by two different types of enzymes [9]: histone acetyltransferases (HATs) and histone deacetylases (HDACs). HATs trigger a reaction that preferentially acetylates specific Lys substrates (histones and nonhistone proteins) to generate a relaxed form of chromatin, which alters gene expressions. HDACs trigger an opposite reaction by removing acetyl groups from Lys substrates to generate a compact form of the chromatin structure [10]. In general, high levels of acetylation of core histones induce highly transcribed genes, and gene silencing was found to be highly correlated with low levels of acetylation [11]. Many studies demonstrated tumor-suppressor gene silencing in cancer [12, 13], and this can be an important therapeutic cancer target. HDAC inhibitors (HDACis) can block deacetylation reactions to maintain a state of high levels of acetylation of core histones to activate silent genes such as *p53*, *p21^(Waf1/Cip1)^*, *RB*, *p16*, *BRCA1*, and *BRCA2* [14–17]. Restoring normal tumor-suppressor gene function is an important strategy in cancer treatment [18]. HDACis can achieve this. Inhibition of HDAC's function can target certain specific genes which result in cell-cycle arrest, and induce either differentiation or apoptosis in several cancer cell lines [19–22]. Other studies demonstrated that HDACis efficiently inhibited tumor growth in human xenograft models and may be developed as novel anticancer agents [23, 24].

Mammalian HDACs are divided into four classes based on their function and structural homologies to yeast HDACs. Rpd3 (class I), Hda1 (class II), Sir2 (class III), and class IV are atypical categories [25]. Class I HDACs include HDAC-1, -2, -3, and -8. Class II HDACs are able to shuttle between the nucleus and cytoplasm, include HDAC-4, -5, -6, -7, -9, and -10. HDAC-6 is a very important enzyme that affects cytoskeletal regulation, cell migration, and cell-cell interactions. It can also regulate several biological processes [26]. To design potent anticancer drugs, one needs to focus on inhibiting class I HDACs, because they are found in the nucleus, affect the tertiary chromatin structure, and alter many gene expressions involved in cancer-cell proliferation, differentiation, and apoptosis [27]. HDACs are implicated in cancer not only for modulating histones but also nonhistone proteins. First, modifications of histone proteins are implicated in epigenetics that may lead to cancers. Secondly, nonhistone proteins (such as p53, *α*-tubulin, HIF-1*α*, and HSP90) are also implicated in several oncogenic pathways. HDACis were found to produce few side effects in patients and to have a high capacity for anticancer activities [28]. Therefore, HDACis are categorized as targeted anticancer drugs. Currently, many potent pan-HDACis (nonspecific HDACis) have been identified and are in clinical trials, such as SAHA, MS-275, LAQ-824, FK-228, PXD-101, valproic acid, and sodium phenylbutyrate [29].

Propolis, a natural resinous product, is collected from various plant sources by honeybees, which use it to seal holes in their honeycombs. It is one of the world's most popular functional foods. Ten propolins (propolins A–J) of the active components were isolated and characterized from Taiwanese green propolis (TGP) by our laboratory. Propolins were shown to be powerful antioxidants with anticancer properties. Propolin G was isolated as a seventh compound from TGP [30], and it was found to induce apoptotic effects via activation of caspases in two different types of brain cancer cell lines. This study used propolin G as a starting material via semisynthesis to develop a novel and potent HDACi and evaluated its anticancer activities *in vitro *and *in vivo*.

## 2. Materials and Methods

### 2.1. Cell Culture and Cell Number Determination

Human MCF-7 breast cancer, rat C6 glioma cells were purchased from the Food Industry Research and Development Institute (Hsinchu, Taiwan). All cell lines were cultured in Dulbecco's modified Eagle's medium (DMEM, Gibco, Grand Island, NY, USA) containing 10% fetal bovine serum (FBS), a 1% dilution of penicillin-streptomycin, and 2 mM glutamine. Cells were maintained at 37°C in a humidified atmosphere of 95% air and 5% CO_2_. Human MDA-MB-231 breast cancer cells were also purchased from the Food Industry Research and Development Institute (Hsinchu, Taiwan). Cells were cultured in L-15 medium (Gibco, Grand Island, NY, USA) containing 10% FBS, a 1% dilution of penicillin-streptomycin, and 2 mM glutamine. Cells were maintained at 37°C in a humidified atmosphere of 100% air. Cells were maintained at 37°C in a humidified atmosphere of 95% air and 5% CO_2_. NBM-HD-1 was dissolved in DMSO (dimethyl sulfoxide) and prepared at a fixed concentration of 10 mg/mL. Cells (1.0 × 10^6^ per dish) were cultured in a 100 mm dish and incubated for 14 h before treatment with DMSO or with various concentrations of NBM-HD-1 (2.5, 5.0, 7.5, and 10.0 *μ*g/mL) for different times. NBM-HD-1 is a small compound (MW 584 Da). When these values were converted to molarities, they were 4.3, 8.5, 12.8, and 17.0 *μ*M, respectively. All of these treatments used various doses of NBM-HD-1. The vehicle (DMSO) in the cell culture medium was at a fixed concentration of 2 *μ*L/mL. Cells were counted and determined by a trypan blue exclusion assay.

### 2.2. Primary Astrocytes

Rat astrocytes were prepared from the cerebral cortexes of 17-day-old embryonic rats. The rat cerebral cortex was dissected and incubated with trypsin at room temperature for 5 min. Brain cortex cells were then mechanically dissociated with a fire-narrowed Pasteur pipette in the culture medium and plated at a density of 2 × 10^6^ cells for 10 mL culture medium in a 100 mm dish. Brain cortex cells were cultured in DMEM containing 10% FBS, a 1% dilution of penicillin-streptomycin solution, and 2 mM glutamine for 6 days. Brain cortex cells were rapidly proliferated. Ninety-five percent of the brain cortex cells were differentiation into astrocytes and expressed GFAP (glial fibrillary acidic protein). GFAP is an intermediate filament protein that was through to be specific biomarker for astrocytes in CNS (central nervous system). Astrocytes (3.0 × 10^5^ per well) were seeded in 6-well plates and incubated for 14 h then treated with NBM-HD-1 at various concentrations for 48 h for PCR assay.

### 2.3. Total HDACs Enzymatic Activity Assay

Total HDACs enzyme activity was determined using the Boc-Lys(Ac)-AMC fluorometric HDAC activity assay kit (BioVision, Mountain View, CA, USA). C6 cells were treated with NBM-HD-1 and sodium butyrate (SB; Sigma, St. Louis, MO, USA) for 48 h. Cells were then collected and nuclear fractions were isolated using a NucBuster extraction kit (71183-3; Novagen, San Diego, CA, USA). Equal amounts of nuclear fraction proteins (30.0 *μ*g) were analyzed using a Fluorometric HDAC Activity Assay Kit (k330-100; BioVision). The fluorescence intensity was measured using a fluorometric reader with excitation at 360 nm and emission at 460 nm.

### 2.4. Confocal Microscopy

MCF-7 cells were cultured on six-well culture plates (3.0 × 10^5^/well) and treated with NBM-HD-1 at a fixed concentration of 17.0 *μ*M, 4.0 mM SB, or with SAHA (Alexis Biochemicals, Plymouth Meeting, PA, USA) as a positive control at 5.0 *μ*M for 24 h. After treatment, cells were fixed with methanol (80%) for 30 min, washed with phosphate-buffered saline (PBS), then treated with Triton X-100 (0.3%, v/v) for 5 min, and washed with PBS. Cells were blocked with 5% bovine serum albumin (BSA) for 1 h and washed with PBS, and then the specific antibodies of anti-Ac-histone 3 (1 : 1000) or antigelsolin (1 : 1000) were added overnight. Cells were washed and 2′ antibodies (1 : 1000; anti-mouse immunoglobulin G- (IgG-)FITC or anti-rabbit IgG-TRITC) were added for 1 h. The morphology of nuclear chromatin was defined by analysis of the DNA-binding dye, DAPI (1.0 mg/mL, 5 *μ*L), and protein expression (gelsolin and Ac-histone 3) levels using a confocal imager (BD, Franklin Lakes, NJ, USA).

### 2.5. Western Blot Assay

C6 and MCF-7 cells (1.0 × 10^6^) on 100 mm dishes were treated with 17.0 *μ*M NBM-HD-1 for various times. After treatment, cells were collected and resuspended in 60 *μ*L lysis buffer (RIPA lysis buffer, Millipore, Temecula, CA, USA). Equal amounts of proteins (30.0 *μ*g) were mixed with 2x sample buffer, then resolved by 12.5% SDS-PAGE and electrotransferred to a PVDF membrane (Millipore, Bedford, MA, USA). Equivalent protein loading was verified by staining the membrane with the reversible dye, amido black (Sigma). This was followed by overnight blocking with a solution composed of 20 mM Tris-HCl (pH 7.4), 125 mM NaCl, 0.2% Tween 20, and 3% BSA. Specific antibodies used were anti-Ac-tubulin, antigelsolin, *β*-actin (1 : 1000 of mouse monoclonal antibodies; Sigma), anti-Ac-histone 4 (1 : 1000 of rabbit polyclonal antibodies; Upstate), anti-PTEN, anti-phospho-PTEN, and anti-phospho-AKT (1 : 1000 dilution of rabbit polyclonal antibodies; Cell Signaling Technology). Proteins were detected by enhanced chemiluminescence (ECL; Amersham Pharmacia Biotech, Amersham, UK).

### 2.6. Gene Expression Analysis

Total RNAs were isolated from C6 cells and rat astrocytes. Cells (3.0 × 10^5^ per well) were seeded in 6-well plates and incubated for 14 h then treated with NBM-HD-1 at various concentrations for 48 h. Cells were lysed, and the total RNA was extracted with an RNeasy Mini kit (Qiagen, Valencia, CA, USA). Complementary (c)DNA was prepared by a First Strand cDNA Synthesis Kit (Toyobo, Osaka, Japan). A multiplex PCR was performed (using the primers that are described in Supplementary Table S1 in Supplementary Material available online at doi:10.1155/2012/781417). After an initial denaturation at 95°C for 1 s, 30 cycles were performed at 95°C for 30 s, 52°C for 30 s, and 72°C for 60 s. The last cycle was followed by a 5 min extension at 72°C.

### 2.7. Analysis of the Cell Cycle

C6 and MCF-7 cells (1.0 × 10^6^/dish) in a 100 mm dish were treated with various concentrations (4.3~17.0 *μ*M) of NBM-HD-1 for 48 h. Cells were trypsinized and collected with ice-cold PBS. Cells were resuspended in 200 *μ*L PBS and fixed by adding 800 *μ*L of iced 100% ethanol then incubated overnight at −20°C. Cell pellets were collected by centrifugation, resuspended in 1 mL of hypotonic buffer (0.5% Triton X-100 in PBS and 1.0 *μ*g/mL RNase A), and incubated at 37°C for 30 min. Then, 1 mL of PI solution (50.0 *μ*g/mL) was added, and the mixture was allowed to stand at 4°C for 30 min. The cellular DNA content was then analyzed by FACScan cytometry (Becton Dickinson).

### 2.8. Xenograft Model Assay

Female BALB/c nude mice (5 weeks old) were injected subcutaneously (5.0 × 10^6^ cells/100 *μ*L/mice) with MDA-MB-231 cells. After 2-3 weeks of treatment, approximately 50 mm^3^ tumors were apparent in all mice. Animals were then allocated at random to one of three groups (*n* = 4). Three groups of nude mice were treated with NBM-HD-1 at 50 and 100 mg/kg/day (through an intraperitoneal injection) respectively, and vehicle control group was treated with DMSO/cremophor. Mice were treated every day for 35 days with NBM-HD-1 or the control vehicle. The tumor weight was calculated after the mice were sacrificed.

### 2.9. Statistical Analysis

Animal test and antiproliferation assay results are presented as the mean ± SD. Student's *t*-test was used to calculate the statistical significance of differences between each group and the control group. Differences were considered statistically significant if *P* < 0.05.

## 3. Results

### 3.1. Semisynthesis of NBM-HD-1

**Figure 1:**
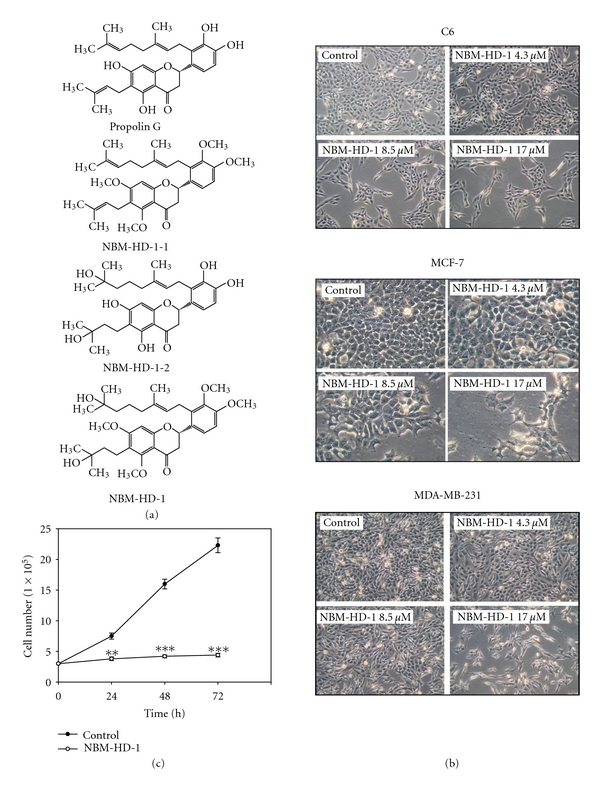
NBM-HD-1 inhibition of cancer cells growth. (a) Structures of propolin G, NBM-HD-1-1, NBM-HD-1-2, and NBM-HD-1. (b) Cells growth inhibition of NBM-HD-1 after treatment with various concentrations in rat C6 glioma cells, human MCF-7 breast cancer cells, and human MDA-MB-231 breast cancer cells for 48 h. (c) Human MDA-MB-231 breast cancer cells (3.0 × 10^5^ per well) were cultured in 6-well plates and incubated for 14 h, then treated with a fixed concentration of 17.0 *μ*M for 1–3 days. Data are shown as the mean ± SD. **P < 0.01; ***P < 0.001 versus the control. All these tests were performed in three independent experiments. A representative experiment of the three replicates is shown.

Propolin G (Figure 1(a)) was isolated through repeated chromatographic runs of the 95% ethanol extract of propolis glue under guidance of brain cancer cell-growth inhibition. Final purification of the active fraction was achieved by HPLC on an RP column. The total content of the active component, propolin G, was roughly 10–12% of the TGP glue [30]. Our previous study suggested that propolin G induced growth inhibition and apoptosis of a brain cancer cell line possibly due to modulation of the expressions of cell cycle-regulator genes and further activation of caspases and mitochondrial pathways, ultimately resulting in the induction of apoptosis in the brain cancer cell lines [30]. Furthermore, we also found that propolin G possesses a weak HDACi property. For this reason, we were interested in the development of a more-potent HDACi from propolin G. Several novel compounds were synthesized and tested (Figure 1(a)). We found that NBM-HD-1 was a very active compound that could inhibit cancer cell growth, due to its potential as an HDACi.

### 3.2. Inhibition of Cell Growth by NBM-HD-1

C6, MCF-7, and MDA-MB-231 cells were treated with NBM-HD-1 at various concentrations of 4.3–17.0 *μ*M for 48 h (Figure 1(b)). These three cancer cell lines were sensitive to NBM-HD-1, with IC_50_ values of 8.5–10.3 *μ*M (Table 1). 

Furthermore, we also evaluated the effect of NBM-HD-1 treatment in various human cancer cell lines including human Hs683 glioma cells, human HT-29 colon cancer cells, and human DBTRG-05MG glioblastoma cancer cells and human Hs68 fibroblast cells (Table 1). All of these cell lines exhibited similar results to those of C6 and MCF-7 or MDA-MB-231 cells. MCF-7, C6, and HT-29 cells were found to be more sensitive than other cancer cell lines. The normal cell line of Hs68 cells was more resistant than other cancer cell lines. These results suggested that NBM-HD-1 is selective for inhibition of different types of cell growth. On the other hand, we also evaluated the inhibition of cell growth activity after treatment of MDA-MB-231 cells with a fixed concentration of 17.0 *μ*M NBM-HD-1 for 24, 48, and 72 h as shown in Figure 1(c). NBM-HD-1 significantly suppressed MDA-MB-231 cell growth in a time-dependent manner. These results demonstrated that NBM-HD-1 possesses the ability to inhibit cancer cell growth. Furthermore, comparison with three different series of analogs of methylated-propolin G (NBM-HD-1-1), hydrated-propolin G (NBM-HD-1-2), and methylated-hydrated-propolin G (NBM-HD-1) was also evaluated for inhibition of the cell-growth capacity (Figure 1(a)). This result demonstrates that methylation or hydration of propolin G alone does not possess excellent activity for cell growth inhibition (data not shown). NBM-HD-1 was derived from propolin G via the two processes of methylation and hydration (Supplementary Material). On the other hand, we also evaluated NBM-HD-1 and propolin G in MDA-MB-231 cells. Treatment with equal concentration (17.0 *μ*M) of compounds, NBM-HD-1 inhibited cell growth and induced differentiation. However, propolin G induced cytotoxicity (data not shown). Taken together, these results suggest that both methylation and hydration are required to obtain an active compound and might be a potent differentiation inducer.

### 3.3. NBM-HD-1 Is an Inhibitor of HDAC

**Figure 2:**
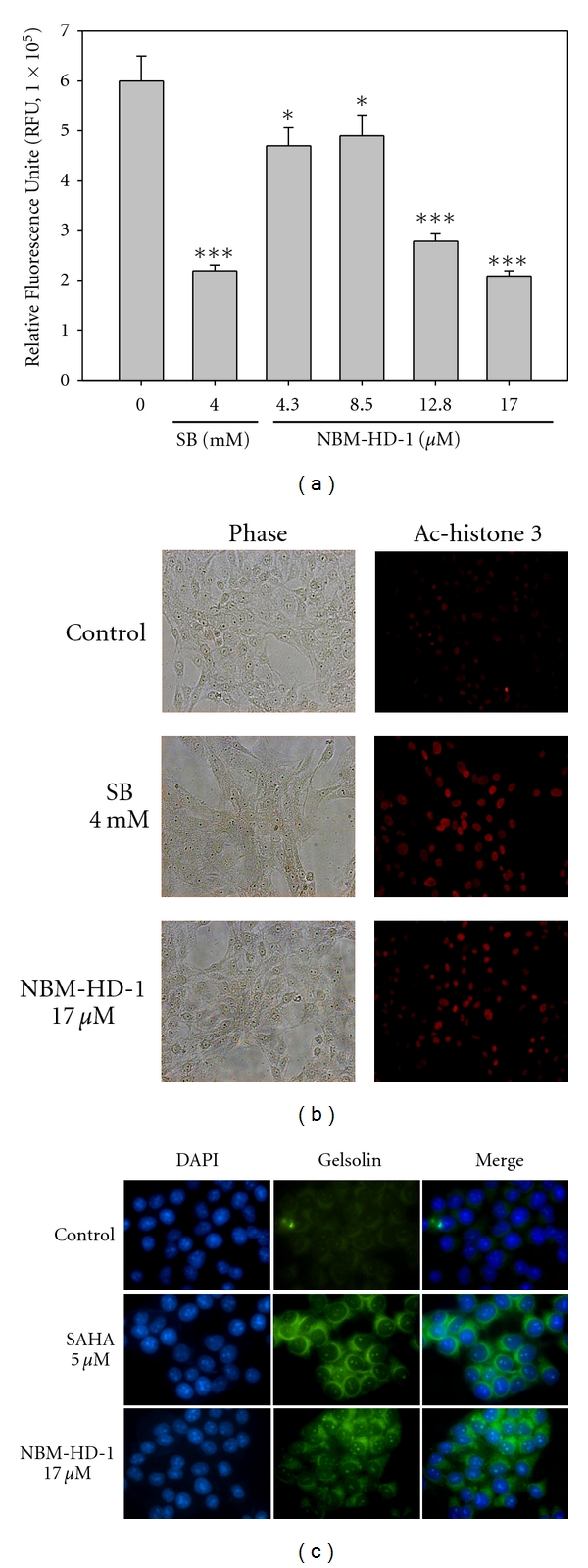
Determination of the inhibition of total HDAC activity by NBM-HD-1. (a) C6 cells (6.0 × 10^5^) were seeded in 60 mm dishes and incubated for 14 h then treated with various concentrations (4.3–17.0 *μ*M) of NBM-HD-1 and SB (4.0 mM, as a positive control) for 48 h, and then the nuclear fraction protein was isolated to determine the inhibition of total HDAC enzyme activity. Data are shown as the mean ± SD. *P < 0.05; ***P < 0.001 versus the control. C6 and MCF-7 cells were cultured in six-well culture plates (3.0 × 10^5^/well) and treated with NBM-HD-1 (at a fixed concentration of 17.0 *μ*M), SAHA (5.0 *μ*M), or SB (4.0 mM) for 24 h. Immunostaining was used to evaluate biomarkers of HDACis such as Ac-histone 3 and gelsolin after cells were treated with HDACis. (b) Ac-histone 3 and (c) gelsolin protein levels were determined by confocal microscopy. All these tests were performed in three independent experiments. A representative experiment of the three replicates is shown.

Using a trypan blue exclusion assay to evaluate cell growth inhibition, we found that NBM-HD-1 significantly inhibited cell growth but not induced cytotoxic effect to those cancer cell lines. Inhibition of cell proliferation was clearly present in NBM-HD-1-treated cells (Table 1 and Figures 1(b) and 1(c)). To determine whether NBM-HD-1 is an inhibitor of HDAC, C6 cells were treated with various concentrations of NBM-HD-1 for 48 h and then nuclear-fraction proteins were isolated to determine the inhibition of HDAC enzyme activity. As shown in Figure 2(a), NBM-HD-1 markedly inhibited nuclear HDACs enzyme activity compared to SB (sodium butyrate, a noted HDACi as a positive control). This result suggests that NBM-HD-1 may be an HDACi. As shown in Figures 2(b) and 2(c), both compounds (SB and SAHA) significantly upregulated Ac-histone 3 and gelsolin protein expressions. Similarly, we also found that NBM-HD-1 significantly increased expression of both proteins (Ac-histone 3 and gelsolin). These results suggest that NBM-HD-1 is an HDACi. 

### 3.4. Upregulation of Ac-Histone 4 Levels by NBM-HD-1

**Figure 3:**
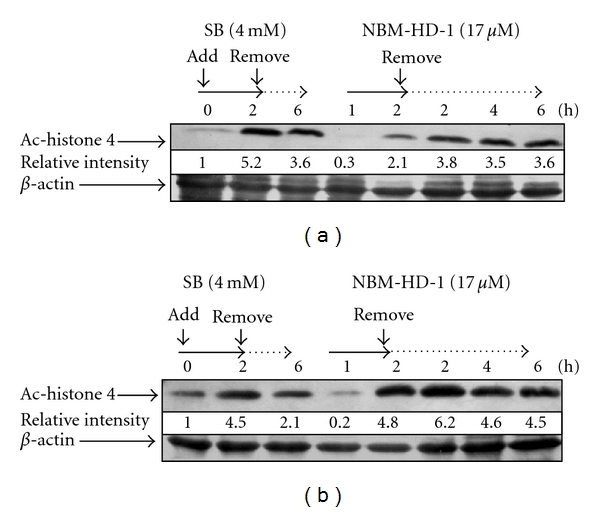
Upregulation of Ac-histone 4 protein expressions after treatment with NBM-HD-1 in various cancer cell lines. (a) C6 cells, and (b) MCF-7 cells were treated with NBM-HD-1 (17.0 *μ*M) or SB (4.0 mM) for 1-2 h or cells were treated with the same concentration of both compounds for 2 h, then the compound was removed, and cells were incubated for another 2, 4, and 6 h. Cell lysates were prepared and subjected to SDS-PAGE and immunoblotting using an Ac-histone 4 antibody.

Next, we examined whether NBM-HD-1 inhibited cell growth through inhibition of HDACs activity in C6 and MCF-7 cells. For this, we used a western blot assay to evaluate whether NBM-HD-1 caused suppression of cell growth through inhibition of HDACs activity. Cells were treated with NBM-HD-1 (17.0 *μ*M) or SB (4.0 mM) and Ac-histone 4 levels significantly increased compared to the untreated group. In this experiment, cells were treated with individual compounds for 2 h as above, then the compounds were removed, and cells were incubated without the drug for another 6 h (SB) or 2, 4, and 6 h (NBM-HD-1). As shown in Figures 3(a) and 3(b), SB-induced Ac-histone 4 protein levels significantly decreased 6 h after removal of SB. In comparison, the decline in Ac-histone 4 protein levels induced by NBM-HD-1 was slower than those induced by SB. Because SB is a very polar compound and can easily dissolve in water, it could very easily be removed from the culture medium. However, the polarity of NBM-HD-1 is less than SB, hence, the water solubility of NBM-HD-1 is relatively less than SB. We assumed that it could easily pass through cell membranes and enter the nucleus. Therefore, after removing NBM-HD-1 from the culture media, cells were still being acted on by NBM-HD-1.

### 3.5. Effects of NBM-HD-1 on Cell-Cycle Regulators

**Figure 4:**
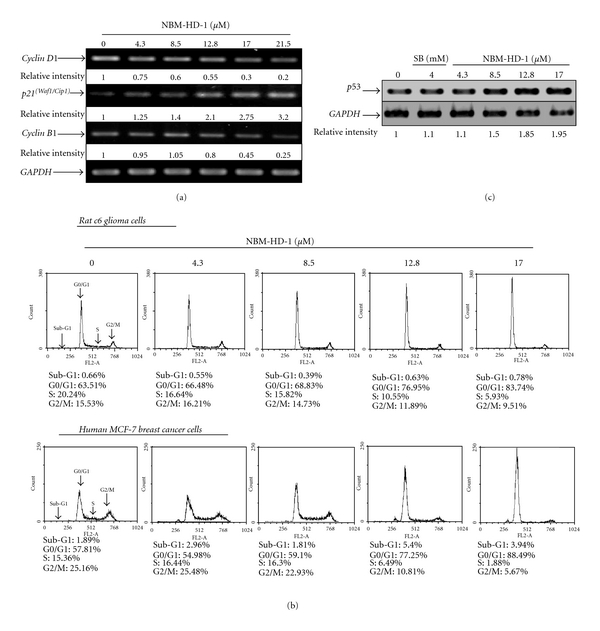
Regulation of cell cycle-regulator and tumor suppressor genes in NBM-HD-1-treated cells. (a) C6 cells (3.0 × 10^5^ per well) were seeded in 6-well plates and incubated for 14 h then treated with NBM-HD-1 at various concentrations (4.3~21.5 *μ*M) for 48 h. *p21^(Waf1/Cip1)^*, *cyclin B1*, and *cyclin D1* cell cycle-regulator genes were determined by RT-PCR. (b) Flow cytometric analysis of NBM-HD-1-treated C6 and MCF-7 cells for 48 h and stained with PI. Following the flow cytometric analysis, the cellular DNA profile was analyzed by Cell Quest software. Rat astrocytes (3.0 × 10^5^ per well) were seeded in 6-well plates and incubated for 14 h then treated with NBM-HD-1 at various concentrations (4.3–17.0 *μ*M) and 4.0 mM of SB for 48 h. (c) *p53 *tumor-suppressor genes were determined by RT-PCR. All these tests were performed in three independent experiments. A representative experiment of the three replicates is shown.

Recently, a study demonstrated that the D1 cyclin and cyclin-dependent kinase inhibitor, p21^(Waf1/Cip1)^, is an important regulator of the cell cycle in breast cancer [31]. Many studies also demonstrated that cell-growth inhibition is regulated by cell-cycle regulators, including the cyclin-dependent kinase inhibitor, p21^(Waf1/Cip1)^, and cyclins B1, D1, and E [32–34]. We investigated the inhibitory effect of NBM-HD-1 on C6 cells growth. Using an RT-PCR, we studied gene expressions of these cell-cycle regulators in C6 cells after treatment with NBM-HD-1 at various concentrations (4.3–21.5 *μ*M) for 48 h. In each case, multiple gene analyses with GAPDH were used to standardize mRNA levels over the course of the experiment. *p21^(Waf1/Cip1)^* gene expressions markedly increased after treatment with NBM-HD-1 at concentrations of 12.8–21.5 *μ*M; while *cyclin B1* and *D1* gene expressions markedly decreased after treatment with NBM-HD-1 at concentrations of 12.8–21.5 *μ*M as shown in Figure 4(a). Several studies demonstrated an association of cancer progression and cell-cycle regulation. Based on these findings, cell-cycle regulation has become a target for controlling cancers [35, 36]. *p21^(Waf1/Cip1)^* is a very important cell-cycle regulator and tumor-suppressor gene. Next, we used flow cytometry to evaluate whether NBM-HD-1-caused cell growth suppression is via regulated cell cycle. C6 and MCF-7 cells were treated with NBM-HD-1 (0–17.0 *μ*M) for 48 h for the cell cycle analysis. Our result indicates no significantly increased apoptotic cell population (sub-G1 phase) following NBM-HD-1 (0–17.0 *μ*M) treatment (Figure 4(b)). However, the cell population at the G0/G1 phase was significantly increased in a dose-dependent manner as shown in Figure 4(b). Taken together, we think that *p21^(Waf1/Cip1)^* gene overexpression may be increased the cell population at the G0/G1 phase after treatment with NBM-HD-1.

### 3.6. Upregulation of Tumor-Suppressor Gene by NBM-HD-1

Rat primary astrocytes were normal brain cells in CNS. Cells were treated with NBM-HD-1 at various concentrations (0~17.0 *μ*M) for 48 h. An RT-PCR was used to measure the tumor-suppressor gene expression. As shown in Figure 4(c), *p53* gene expressions were significantly upregulated after treatment with NBM-HD-1 compared to SB (4.0 mM). This result demonstrates that NBM-HD-1 may have had the capability to restore or awaken tumor-suppressor genes. HDACis are thought to be capable of waking up silenced expression of tumor-suppressor genes [37]. Our results suggest that NBM-HD-1 may be useful in controlling cancer-cell proliferation and maintaining or upregulating the function of tumor-suppressor genes in normal cells.

### 3.7. NBM-HD-1 Inhibits HDAC Enzymatic Activity, Also Suppresses the Phosphorylation of PTEN and AKT

**Figure 5:**
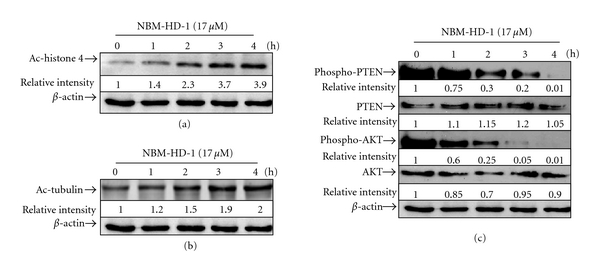
NBM-HD-1 inhibits cell growth through a PTEN/AKT-dependent pathway. MCF-7 cells (1.0 × 10^6^) were seeded in 100 mm dishes, incubated for 14 h, and then treated with NBM-HD-1 at a fixed concentration of 17.0 *μ*M for 1, 2, 3, and 4 h. Cell lysates were prepared and subjected to SDS-PAGE and immunoblotting using respective specific antibodies. (a) Ac-histones 4. (b) Ac-tubulin. (c) Phospho-PTEN and phospho-AKT. HDACi biomarkers were determined by a western blot assay.

This study also explored the anticancer mechanisms of NBM-HD-1. The PI3K pathway plays a key role in cell-signal transduction. AKT is the downstream target of PI3K in controlling angiogenesis and tumor growth. It can regulate many cell functions such as proliferation, transformation, apoptosis, tumor growth, and angiogenesis. Therefore, controlling the PTEN or AKT signal pathway may be an important anticancer mechanism. Cells were treated with NBM-HD-1 at a fixed concentration of 17.0 *μ*M for 1, 2, 3, and 4 h. As shown in Figure 5(a), protein levels of Ac-histones 4 markedly increased after treatment for 2–4 h. Similar result was also seen with Ac-tubulin protein as shown in Figure 5(b). These results demonstrate that NBM-HD-1 may be able to rapidly move into cell membranes and the nucleus to regulate chromatin remodeling and affect gene and protein expressions. We also found that NBM-HD-1 suppressed phospho-PTEN expression after treatment for 2 h (Figure 5(c)). A similar result was also found for the phospho-AKT protein as shown in Figure 5(c). Neither PTEN nor AKT expression markedly changed after treatment with NBM-HD-1. Taken together, our data suggest that NBM-HD-1 not only inhibits HDAC enzyme activity but also downregulates PTEN and AKT phosphorylation.

### 3.8. NBM-HD-1 Inhibits Tumor Growth in Mice

**Figure 6:**
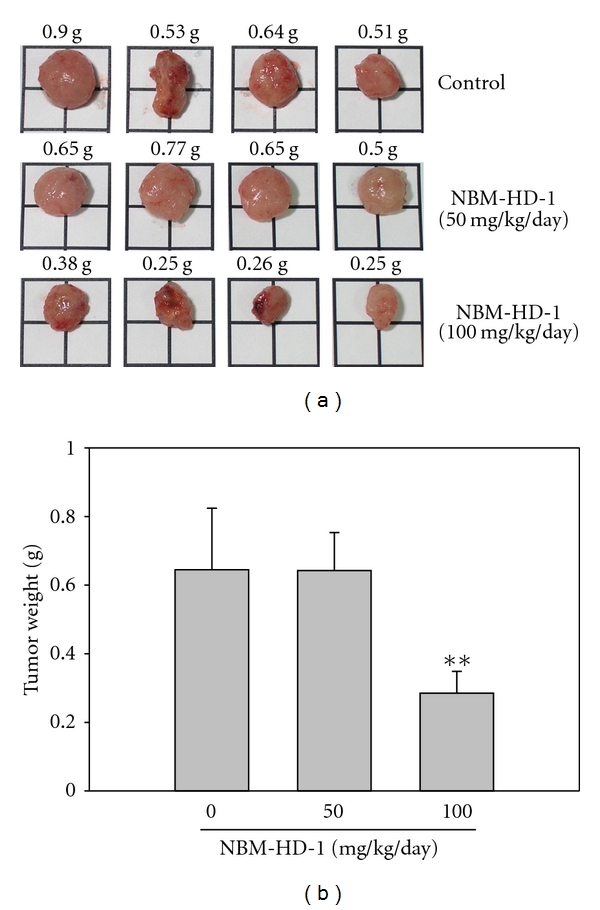
NBM-HD-1 inhibits tumor growth *in vivo *after intraperitoneal (i.p.) administration. Nude mice were injected subcutaneously with MDA-MB-231 cells (5.0 × 10^6^ per mouse). NBM-HD-1 was i.p. injected daily for 35 days. (a) The surgical tumors. (b) Tumor weights were measured after the mice were sacrificed. Student's *t*-test was used to calculate the statistical significance of differences between each group and the control group. Data are shown as the mean ± SD. **P < 0.01 versus the control.

NBM-HD-1 was injected intraperitoneally (i.p.) into immunodeficient mice once daily for 35 days. As shown in Figure 6(a), the tumor size significantly decreased after treatment with NBM-HD-1 (100 mg/kg/day). Furthermore, tumor weights were calculated and analyzed by Student's *t*-test as shown in Figure 6(b). The body weight of mice did not significantly change after treatment with NBM-HD-1 (data not shown). The activity of NBM-HD-1 in this MDA-MB-231 cells xenograft model suggests that it may be a good lead compound for developing novel HDACi for targeted cancer therapy.

## 4. Discussion

In the present study, we have described a novel type of HDACi with potent antiproliferative activity in several cancer cell lines (Table 1). We also showed that an i.p. injection of NBM-HD-1 exhibited antitumor activity in an MDA-MB-231 breast cancer xenograft model (Figure 6). Currently, several HDACis that have come up with predictable treatment for hematological malignancies are still in developmental stage [20, 38]. The US Food and Drug Administration approved SAHA (vorinostat) and FK-228 (romidepsin) for treating cutaneous T-cell lymphoma (CTCL) in 2006 and 2009, respectively. However, HDACis activity in solid tumors has been limited [39]. The reason why HDACi activity in solid tumors is weak compared to hematological malignancies may be its correlation with the inhibition classes of HDACs by HDACi with different cancer cells.

 SAHA is one of the most promising pan-HDACi which can potently inhibit several HDACs such as HDAC-1, -2, -3, -6, -8, and -10 in the nanomolar range [40]. In particular, SAHA inhibits class I HDACs (HDAC-1, -2, and -3), resulting in the acetylation of nuclear histone proteins which affects the chromatin structure. Finally, SAHA influences the expressions of many genes involved in cancer-cell proliferation, apoptosis, and differentiation. However, one study demonstrated that HDACi's inhibition of HDAC-1 may trigger neurotoxicity [41]. In our laboratory, we found that a low dose of about 1.0 *μ*M of SAHA had strong neurotoxicity on rat cortical neurons and neural stem cells, but this phenomenon did not exist by treatment with NBM-HD-1 (data not shown). These results support the current trend towards developing specific HDACis for targeted cancer therapy with reduced side effects. 

Propolin G is a natural product isolated and characterized from TGP. Previously, we showed that propolin G possesses a strong capacity to induce apoptosis in brain cancer cells [30]. We also found that it can pass the blood-brain barrier (BBB, data not shown). However, developing potent HDACis with few side effects is very important for the acceptance of these agents for targeting therapeutic drugs. For this reason, NBM-HD-1 was derived through a two-step semisynthesis process. Several NBM-HD-1 analogs were generated and tested in antiproliferation assays in several cancer cell lines. Among them, NBM-HD-1 had an excellent capacity to inhibit cell growth. The result suggests that both hydration positions at geranyl and isoprenyl side chains are required to obtain an active compound. Furthermore, methylation of these compounds are also required for anticancer activity. Compare to our previous study [42], we demonstrated that either three methoxy groups (3′,4′,7-trimethoxyflavanone, NBM-HD-3) or four methoxy groups (3′,4′,5,7-tetramethoxyflavanone, NBM-HD-1) exhibited highly inhibition activity of HDACs. We use different methods to synthesis these two compounds while both of them are derived from Taiwanese green propolis. Actually, some major differences existed among these two compounds. First, NBM-HD-3 is effective for antiproliferation on the brain tumor cells [42]; however, NBM-HD-1 is more pronounced in targeting human breast cancer cells as shown in Table 1 and Figure 6. For example, in MCF-7 human breast cancer cells, the IC_50_ in NBM-HD-3 is 40% higher than NBM-HD-1. On the contrary, in Hs683 human brain cancer cells, NBM-HD-1 is almost half potency compared to NBM-HD-3. Second, the translation regulation of PTEN and AKT proteins by these two compounds is quite different. After treatment with NBM-HD-3, the expression of either PTEN or AKT proteins are elevated significantly [42], but not in the cells that treatment with NBM-HD-1 (Figure 5(c)). Third, it has been demonstrated that the flavonoids with 5-methoxy group has lower antioxidant activities then those with 5-hydroxy group and showed relatively low IC_50_ to cancer cells [43]. This may indicate that the compound NBM-HD-1 may exhibited relatively low antioxidant activity compared with NBM-HD-3. However, the results we observed from these two semisynthetic compounds showed that the effective antitumor ability is provided with NBM-HD-1 and NBM-HD-3 against breast cancer and brain cancer, respectively. On the other hand, several classes of HDACis were defined [44], including (a) short-chain fatty acids (SB, sodium phenylbutyrate, and valproic acid); (b) cyclic tetrapeptides (trapoxin, FK-228, and apicidin); (c) hydroxamic acids (TSA, SAHA, and LAQ-824); (d) benzamides (MS-275). NBM-HD-1 could not be classified into any of these four classes. Therefore, it is a novel type of HDACi.

From the western blot data, we found that NBM-HD-1 had an effect on cancer cells within a short period after treatment with a fixed concentration of 17.0 *μ*M at 1–4 h as shown in Figure 5. Ac-histone 4 protein levels markedly increased suggesting that NBM-HD-1 might affect HDAC-3 and increase Ac-histone 4 expressions in nuclei of cancer cells. Ac-tubulin significantly increased after treatment with NBM-HD-1. This result suggests that NBM-HD-1 can affect HDAC-6. Interestingly, phosphorylations of PTEN and AKT were markedly downregulated after treatment with NBM-HD-1. This result suggests that NBM-HD-1's inhibition of cancer-cell growth may be via downregulation of the PTEN/AKT signal pathway. Based on these findings, we speculated that the PTEN/AKT pathway is involved in NBM-HD-1's induction of cancer-cell growth inhibition in MCF-7 cells. Taken together, our data suggest that NBM-HD-1 not only inhibits HDACs enzyme activity but also suppresses the PTEN/AKT pathway in cancer cells.

Concluding this study, we obtained a novel HDACi, called NBM-HD-1, derived from propolin G using two simple steps (methylation and hydration, Supplementary Material). Our data suggest that NBM-HD-1 may be a potent HDACi which suppresses cancer-cell growth in several cancer cell lines. This anticancer effect did not occur through a cytotoxic effect (Figures 1(b) and 4(b)). Its anticancer mechanisms may involve two pathways. First, NBM-HD-1 inhibited HDAC enzyme activity, affecting chromatin core histones and induced changes in gene expression to control the cell cycle. Second, NBM-HD-1 suppressed the PTEN/AKT pathway to inhibit cancer cell growth. These two factors may be pertinent to the anticancer effect of NBM-HD-1 treatment. Developing promising HDACis for treating solid tumors is a difficult problem. NBM-HD-1 seems to have the potential to resolve this problem.

##  Funding

The paper was supported by Grants from the National Science Foundation Directorate for Engineering of Taiwan (SBIR, 1Z-960165) and the National Science Council of Taiwan (NSC97-2320-B-038-010-MY3).

##  Conflict of Interests

No potential conflict of interests were disclosed.

## Supplementary Material

NBM-HD-1 was derived from propolin G via the two processes of methylation and hydration. The steps of extraction, isolation of Propolin G from Taiwanese Green Propolis (TGP), and the processes of methylation and hydration from Propolin G are available in the supplementary material.

## Figures and Tables

**Table 1:** Inhibition of cancer cell lines by NBM-HD-1. Cells were incubated with NBM-HD-1 for 3 days.

Cell lines	IC_50_ (*μ*M)
MCF-7	8.5
C6	8.7
MDA-MB-231	10.3
Hs683	12.7
HT-29	9.4
DBTRG-05MG	12.3
Hs68 (normal cell line)	25.0
