# Supplementary material for: NBM-HD-1: A Novel Histone Deacetylase Inhibitor with Anticancer Activity
Source: Evid Based Complement Alternat Med. 2011 Oct 20;2012:781417. doi: 10.1155/2012/781417 (PMC3199191; doi:10.1155/2012/781417)
Supplement: Supplementary file 1 — NBM-HD-1 was derived from propolin G via the two processes of methylation and hydration. The steps of extraction, isolation of Propolin G from Taiwanese Green Propolis (TGP), and the processes of methylation and hydration from Propolin G are available in the supplementary material. [file 781417.f1.doc]

**Supplementary Materials and Methods**

**Extraction and isolation of Propolin G (1)**

TGP (200 g) was extracted with 95% ethyl alcohol (2000 mL ×3), sonicated for 3 h, and left to stand for 7 days at 25°C. The filtered ethanol extract was evaporated to dryness under reduced pressure to yield a brown gum (150.2 g), which was kept at -20°C until used. A portion of this extract (10.0 g) was fractionated over a Sephadex LH-20 column (Amersham Pharmacia Biotech AB, Uppsala, Sweden) using methanol as the solvent to elute and furnish six fractions. All eluates, including fractions from the follow-up chromatographic runs, were assayed for brain cancer cell growth inhibition, and the active fractions were chromatographed again on a silica gel column using a gradient solvent system of *n*-hexane and EtOAc for elution. Purification of the most active fraction 3 (*n*-hexane: EtOAc, 70: 30) was carried out on a reversed-phase (RP) preparative high-performance liquid chromatography (HPLC)/ultraviolet (UV) unit. Fractions at retention times of 25.0 min corresponding to propolin G were collected. Conditions were as follows: a Luna Phenomenex column (C_18_, 250 × 10 mm); a solvent system of methanol: water (8.5: 1.5); a flow rate of 3.5 mL/min; and detection at UV 280 nm. We identified the compound as propolin G (Fig. 1). The purity of the propolin G was estimated to be no less than 95% by HPLC/UV based on the peak area.

**Preparation of 3',4',5,7-O-tetramethyl Propolin G (2)**

To a solution of propolin G (**1**) (152 mg, 0.31 mmol), K_2_CO_3_ (431 mg, 3.1 mmol) and acetone (15 mL) were added with Me_2_SO_4_ (0.25 mL, 2.48 mmol), and the resulting solution was heated under N_2_ for 24 h. After removal of the organic solvent, the residue was dissolved in CH_2_Cl_2_ (50 mL) and washed with distilled (dis)-H_2_O (50 mL x3). The organic layer was dried over Na_2_SO_4_ and evaporated under reduced pressure. The residue obtained was purified by a silica gel column (EtOAc: *n*-hexane = 1: 6) to give **2** (127 mg, yield: 75%). ^1^H-NMR (400 MHz, CDCl_3_)  7.26 (1H, d, J=8.6 Hz), 6.86 (1H, d, J=8.6 Hz), 6.27 (1H, s), 5.50 (1H, dd, J=2.5, 13.5 Hz), 5.14-5.11 (1H, m), 5.11-5.10 (1H, m), 5.02-4.99 (1H, m), 3.87 (3H, s), 3.83 (3H, s), 3.79 (6H, s), 3.50 (1H, dd, J=6.6, 15.2 Hz), 3.43 (1H, dd, J=5.8, 15.2 Hz), 3.00 (1H, dd, J=13.5, 16.7 Hz) , 2.68 (1H, dd, J=2.6, 16.7 Hz), 2.00-1.92 (2H, m), 1.75 (3H, s), 1.70 (3H, s), 1.65 (3H, s), 1.60 (3H, s), 1.52 (3H, s); ^13^C-NMR (100 MHz, CDCl_3_)  189.4 (s), 164.0 (s), 163.3 (s), 159.6 (s), 153.0 (s), 147.2 (s), 135.6 (s), 134.2 (s), 131.5 (s), 131.3 (s), 129.8 (s), 124.1 (d), 122.8 (d), 122.7 (d), 122.1 (d), 118.2 (s), 110.3 (d), 108.7 (s), 95.6 (d), 75.9 (d), 61.8 (q), 60.7 (q), 55.7 (q), 55.7 (q), 45.1 (t), 39.6 (t), 26.6 (t), 25.7 (q), 25.6 (q), 24.9 (t), 22.0 (t), 17.7 (q), 17.6 (q), 16.3 (q); HREIMS *m/z* 548.3140 (calcd. for C_34_H_44_O_6_ 548.3142).

**Preparation of 6-(3-hydroxy-3-methylbutyl)-2’-(7-hydroxy-3,7-dimethyloct-3-enyl)-3',4',5,7-tetramethoxyflavanone (3)**

To a solution of **2** (200 mg, 0.37 mmol) in THF (6 mL) was added 49% H_2_SO_4_ (4 mL) in a dropwise manner in an ice bath. The reaction mixture was then stirred at room temperature (RT) for 8 h and diluted with H_2_O. The aqueous layer was extracted with CH_2_Cl_2_ (50 mL x3). The combined organic layers were dried over Na_2_SO_4_ and evaporated under reduced pressure to give a residue, which was purified by a silica gel column (EtOAc: *n*-hexane 1: 6~1: 1) to give the pure oil **3** (54 mg, yield: 25%). IR (KBr) *ν*_max_ cm^-1^ 3745, 3443, 2968, 1676, 1600, 1455; ^1^H-NMR (400 MHz, CDCl_3_)  7.27 (1H, d, J=8.6 Hz), 6.87 (1H, d, J=8.6 Hz), 6.28 (1H, d, J=2.6 Hz), 5.49 (1H, dd, J=2.4, 13.6 Hz), 5.05 (1H, t, J=6.0 Hz), 3.87 (3H, s), 3.84 (3H, s), 3.79 (6H, s), 3.52 (1H, dd, J=5.5, 15.2 Hz), 3.43 (1H, dd, J=7.6, 15.2 Hz), 2.97 (1H, dd, J=8.0, 16.7 Hz), 2.70-2.59 (3H, m), 1.93 92H, t, J=6.4 Hz), 1.65 (3H, s), 1.64-1.60 (2H, m), 1.41-1.32 (4H, m), 1.25 (6H, s), 1.15 (3H, s), 1.14 (3H, s); ^13^C-NMR (100 MHz, CDCl_3_)  189.4 (s), 164.0 (s), 163.3 (s), 159.6 (s), 153.0 (s), 147.2 (s), 135.6 (s), 134.2 (s), 131.5 (s), 131.3 (s), 129.8 (s), 124.1 (d), 122.8 (d), 122.7 (d), 122.1 (d), 118.2 (s), 110.3 (d), 108.7 (s), 95.6 (d), 75.9 (d), 61.8 (q), 60.7 (q), 55.7 (q), 55.7 (q), 45.1 (t), 39.6 (t), 26.6 (t), 25.7 (q), 25.6 (q), 24.9 (t), 22.0 (t), 17.7 (q), 17.6 (q), 16.3 (q); HREIMS *m/z* 584.3344 (calcd. for C_34_H_48_O_8_ 584.3338).
