# Supplementary material for: NBM-HD-1: A Novel Histone Deacetylase Inhibitor with Anticancer Activity
Source: Evid Based Complement Alternat Med. 2011 Oct 20;2012:781417. doi: 10.1155/2012/781417 (PMC3199191; doi:10.1155/2012/781417)
Supplement: Supplementary file 2 [file 781417.f2.doc]

**Table S1**. Primer sequences

| **Gene name** | **Sequence** |
| --- | --- |
| p21^(Waf1/Cip1)^ | forward 5’-GGGGGCATCATCAAAAACTT-3’ |
|  | reverse 5’-ACTGAAGGGAAAGGACAAGG-3’ |
| cyclin B1 | forward 5’-CGGGAAGTCACTGGAAACAT-3’ |
|  | reverse 5’-AAACATGGCAGTGACACCAA-3’ |
| cyclin D1 | forward 5’-CTGGCCATGAACTACCTGGA-3’ |
|  | reverse 5’-GTCACACTTGATCACTCTGG-3’ |
| p53 | forward 5’-GCCATCTACAAGAAGTCACA-3’ |
|  | reverse 5’-GTCTTCCAGCGTGATGATG-3’ |
| GAPDH | forward 5’-CCCATCACCATCTTCCAG-3’ |
|  | reverse 5’-CAGTCTTCTGGGTGGCAGT-3’ |
